# App-Based Delivery of Clinical Emotional Freedom Techniques: Cross-Sectional Study of App User Self-Ratings

**DOI:** 10.2196/18545

**Published:** 2020-10-14

**Authors:** Dawson Church, Peta Stapleton, Debbie Sabot

**Affiliations:** 1 National Institute for Integrative Healthcare Fulton, CA United States; 2 School of Psychology Faculty of Society & Design Bond University Robina Australia

**Keywords:** anxiety, stress, meditation, mobile health, Emotional Freedom Techniques (EFT), mobile phone

## Abstract

**Background:**

The burgeoning area of mobile health (mHealth) has experienced rapid growth in mobile apps designed to address mental health issues. Although abundant apps offer strategies for managing symptoms of anxiety and stress, information regarding their efficacy is scarce.

**Objective:**

This study aimed to assess the effect of an mHealth app on user self-ratings of psychological distress in a sample of 270,461 app users. The Tapping Solution App guides users through the therapeutic protocols of Clinical Emotional Freedom Techniques (EFT), an evidence-based psychophysiological intervention that combines acupressure with elements of cognitive and exposure therapies.

**Methods:**

App users provided self-ratings of emotional intensity before and after app sessions (termed “tapping meditations”) using an 11-point Subjective Units of Distress scale. App user data for 23 tapping meditations, which addressed psychological symptoms of anxiety and stress, were gathered between October 2018 and October 2019, totaling 380,034 completed app sessions.

**Results:**

Across 12 anxiety-tapping meditations, the difference in emotional intensity ratings from presession (mean 6.66, SD 0.25) to postsession (mean 3.75, SD 0.30) was statistically significant (*P*<.001; 95% CI −2.92 to −2.91). Across 11 stress-tapping meditations, a statistically significant difference was also found from presession (mean 6.91, SD 0.48) to postsession (mean 3.83, SD 0.54; *P*<.001; 95% CI −3.08 to −3.07). The results are consistent with the literature on the efficacy of Clinical EFT for anxiety and stress when offered in conventional therapeutic formats.

**Conclusions:**

The findings provide preliminary support for the effectiveness of the mHealth app in the immediate reduction of self-rated psychological distress. As an adjunct to professional mental health care, the app promises accessible and convenient therapeutic benefits.

## Introduction

### Background

Symptoms of anxiety and psychological distress are highly prevalent in the adult population worldwide. Anxiety disorders are among the most common mental health disorders [1], with an estimated one-third of the global population affected by an anxiety disorder during their lifetime [2,3]. Furthermore, subclinical symptoms of anxiety are reported globally, which can significantly impair functioning and reduce quality of life [4]. Psychological stress is also a commonly reported mental health issue. More than 75% of American adults perceive themselves as significantly stressed, with 42% expressing a desire to manage their stress [5].

Chronic exposure to stress is associated with enduring changes in the body’s emotional, physiological, and behavioral responses [6]. These changes present an increased risk for mental health disorders and diseases such as depression, cardiovascular disease, autoimmune dysfunction, and cancer [7]. Several pathways link psychological distress to disease. Maladaptive behavioral changes in response to stress (eg, inadequate sleep, poor diet) and biological changes in the endocrine response system (eg, hypothalamic-pituitary-adrenocortical axis, sympathetic-adrenal-medullary) can increase individuals’ risk of disease [8,9]. As a result, adverse psychological health imposes a substantial economic burden at individual and societal levels [10,11]. The very high levels of anxiety and psychological stress reported globally highlight the need for accessible psychological treatments with demonstrated efficacy to help reduce the behavioral and biological outcomes associated with poor mental health [7].

### Mobile Health

Mobile technology innovation has significantly transformed aspects of everyday life. This technological platform has altered the way we consume entertainment, educate ourselves, and communicate with each other [12] by broadening access to services and increasing resource availability. Mobile technologies in the field of mental health have the potential to revolutionize traditional health care [13]. Mobile health (mHealth) is an emerging field in psychological health practice that uses wireless technologies supported by smartphones and mobile devices [14]. mHealth apps are considered as a new taxonomy of techniques that help to manage psychological distress [15] and enable users to work independently on aspects of self-improvement. As an adjunct to psychotherapy, mHealth apps offer increased access and availability than face-to-face health care [16].

The burgeoning growth in mental health apps has been largely attributed to rapid technological development, together with its convenience and ease of use [17,18]. Although mHealth apps (eg, medical, health, and fitness categories) currently account for 3.4% of the 3 million plus apps available via the Apple Store, an estimated 60% of smartphone users in the United States have at least one mHealth app installed on their smartphones [19]. There is an abundance of commercial apps offering users strategies and techniques for managing anxiety and stress. However, little information on the efficacy of these apps is available beyond self-rated reviews and star ratings [20]. The proliferation of mobile apps to address mental health calls for timely evaluation of their psychological benefits for app users.

### Apps Designed for Anxiety

Several recent meta-analyses have comprehensively appraised the field of mHealth apps with particular focus on apps designed to manage symptoms of anxiety. Sucala et al [21] analyzed 52 apps available through European app stores (iTunes and Google Play), which targeted anxiety in general, worry, and/or panic attacks. In 63.5% of the apps analyzed, no information was given to users about the therapeutic method that informed its design. Of the apps that identified a therapeutic method, 26.9% were aligned with cognitive behavioral therapy, while 7.7% reported a combination of therapies (eg, meditation, mindfulness, neuro-linguistic programming). The majority of apps failed to disclose details regarding professional licensure and developer training. Of the 52 anxiety-based apps reviewed, Sucala et al [21] identified only two studies that presented feasibility and efficacy data [22,23], both of which suggested that the apps effectively reduced symptoms of anxiety. However, both the respective studies had notable limitations regarding their research design (eg, lack of empirically validated measures or high participant attrition). Accordingly, Sucala et al [21] recommended that the anxiety app design be grounded in psychotherapeutic techniques with demonstrated efficacy in face-to-face clinical settings. Furthermore, they cautioned that apps not grounded in empirical approaches can result in iatrogenic effects, thereby increasing symptoms of anxiety in app users.

The efficacy of anxiety-based smartphone-supported apps was also examined in a meta-analysis of 9 randomized controlled trials (RCTs) that met specific systematic review criteria [24]. Collectively, a significant reduction in anxiety symptoms was found in the anxiety app intervention groups compared with controls (N=1837), with the greatest benefits observed in the trials that compared smartphone interventions with wait list control conditions. Although significantly smaller effects were observed in studies that controlled for attention or user engagement, Firth et al [24] concluded that smartphone interventions appeared to reduce anxiety symptoms significantly more than controls. However, there was substantial variance in anxiety levels between and within study participants, indicating the need for research to identify specific user groups who may benefit most from anxiety interventions delivered via mobile device platforms.

### Apps Designed for Stress

Apps designed to help users manage psychological stress have also been the subject of meta-analytic review. In 2016, Coulon et al [25] provided the first meta-analysis of evidence-based stress management apps. A total of 902 apps available on the Apple iOS platform were subject to a multilevel selection process, of which 32 apps met 3 specific criteria: domains related to evidence-based content, transparency of app developer details, and functional app interface. The most common therapeutic techniques among the apps were mindfulness, meditation, and diaphragmatic breathing. Several apps purported to deliver efficacious stress management techniques (eg, breathing techniques), despite providing inadequate guidance for users (eg, lack of instruction regarding the use of diaphragm muscles during breathing exercises). Therefore, Coulon et al [25] cautioned that apps delivering evidence-based methods require adequate behavioral skill instruction to avoid iatrogenic effects on app users.

Coulon et al [25] were the first to apply an established taxonomy of behavior change techniques [26] in the review of stress management apps. In extending the research of Coulon et al [25], Christmann et al [15] proposed an additional taxonomy of emotion-focused stress management strategies in their review of free stress management apps available on Google Play. Of the 62 apps that met their inclusion criteria, 26 apps comprised behavioral change–based strategies and 15 apps presented emotion-focused stress management techniques. One app was common to both the analyses by Christmann et al [15] and Coulon et al [25]. In contrast with the review by Coulon et al [25], in which 48% of stress management apps drew on mindfulness or meditation techniques, only one-third (34%) of apps reviewed by Christmann et al [15] used empirically demonstrated approaches (eg, meditation, mindfulness, breathing, acupressure or EFT). The standardized taxonomy proposed by Christmann et al [15] was designed to enable greater comparability between different intervention types in stress management apps. Interestingly, although some apps reviewed by Christmann et al [15] offered users the opportunity to self-rate symptoms and stress levels, none used that information to address the pattern of self-rated symptoms within app content. They considered this as an important area for future health app design. This self-rating functional aspect of app design is addressed in this study.

Meta-analytic findings provide direction for future mHealth app assessment and development. Collectively, this body of work reinforces a crucial principle: mHealth apps must demonstrate positive outcomes for app users [27]. However, few studies have examined the effectiveness of mHealth apps in reducing symptoms of psychological distress [12]. The mHealth platform offers the potential for a range of self-management strategies to assist psychological symptoms of anxiety and stress, particularly for individuals who require psychological support but have limited access to regular health care [24,28]. As the mHealth app modality offers benefits such as increased flexibility, accessibility, convenience, and reduced cost [12,29], studies that examine the effectiveness of evidence-based apps are paramount to help inform and protect the growing population of app users.

### The Tapping Solution App

The Tapping Solution App, developed by The Tapping Solution, LLC, is an Energy Psychology–based meditation app for use on smartphones and mobile devices. The app was designed to improve users’ symptoms of psychological distress (eg, anxiety, stress, worry) and promote overall well-being using Emotional Freedom Techniques (EFT). EFT is a therapeutic approach in the field of Energy Psychology, which combines elements of exposure and cognitive therapy together with somatic stimulation. In the EFT therapeutic protocol, the individual taps with the fingertips on specific acupoints on the body (acupressure) while focusing on cognitions that produce emotional distress [30]. This focus on emotionally charged memories and beliefs draws from the field of exposure therapy. When paired with acupoint tapping, the emotional intensity of these memories is usually quickly reduced [31]. Since its inception in the 1990s, EFT has been a manualized therapy, leading to uniformity of application in research and training. The manualized form of the method is called Clinical EFT.

**Figure 1:**
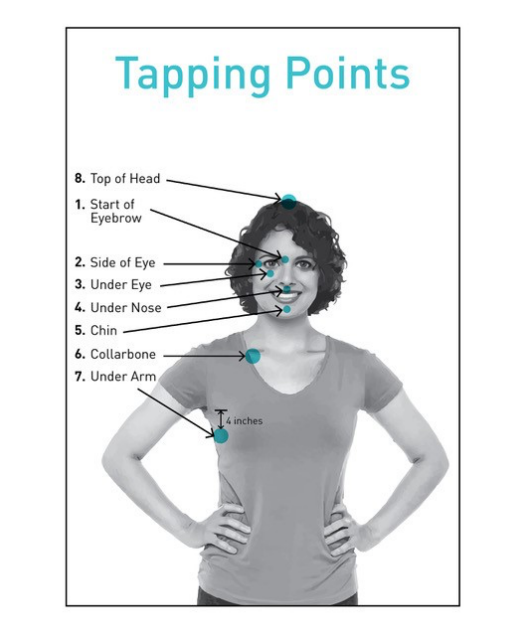
Eight tapping points utilized in Emotional Freedom Techniques practice. The Tapping Solution App includes a point on the side of the hand. Copyright 2019 by Peta Stapleton. Reprinted with permission.

The EFT procedure begins with clients identifying an issue and rating their degree of distress. The EFT uses an 11-point Likert scale ranging from 0 (no emotional intensity) to 10 (maximum emotional intensity). This scale is called the Subjective Units of Distress (SUD) scale and originates in the work of Wolpe [32]. Clients provide a phrase that encapsulates their issue, such as “the car crash” or “the explosion.” This “reminder phrase” is repeated throughout treatment to maintain and reinforce exposure to the issue while the acupoints are stimulated. A long form of EFT includes eye movements similar to those used in eye movement desensitization and reprocessing [33] and stimulates 14 acupoints. An abbreviated version stimulates 9 points (8 points are displayed in Figure 1 [34], excluding the side of the hand acupoint). The EFT short form is completed in less than 30 seconds and is referred to as a “round” of tapping. The procedure is repeated until the SUD levels drop, which may require several rounds.

The psychological benefits of EFT intervention include improvements in symptoms of anxiety, posttraumatic stress disorder (PTSD), self-esteem, and pain [31,35-39]. Other studies have reported rapid improvements for a variety of additional psychological challenges such as performance blocks, social anxiety, excessive food cravings, and stress management [31,37,38,40-43]. A web-based research bibliography listing more than 100 clinical trials is publicly available [44].

In the 1990s, Division 12 (Clinical Psychology) of the American Psychological Association published standards for “empirically validated therapies” [45,46]. For the next two decades, the principles guided the design and reporting of EFT research [31]. Several studies have examined symptom levels before and after a single session of EFT (≤60 min in duration). These studies showed that EFT is effective for fear of public speaking [39,40,47], sports performance [48,49], anxiety and depression [50], phobic fear [51-53], and traumatic stress [54,55].

Although this study is the first app-based EFT study, it is important to note that several other studies have examined EFT delivered remotely. Hartung and Stein [56] compared the telephone delivery of EFT with in-person therapy. Although face-to-face delivery of EFT was significantly more efficacious than the telephone (91% vs 67% recovery rate), remote telephone sessions nonetheless remediated PTSD symptoms in 67% of the veterans treated. In a web-based EFT intervention of patients with fibromyalgia, Brattberg [57] found significant improvements in pain and other symptoms. Fibromyalgia was resolved in approximately one-third of the participants and another third reported partial pain relief. In addition, Church and Clond [58] compared participants in a web-based relationship class with a similarly sized sample taking the same class in-person. Although the relationship satisfaction outcomes were similar for both groups, they differed significantly on baseline measures of anxiety, depression, and relationship satisfaction. The authors suggested that the demographic and mental health characteristics of those seeking web-based treatment may differ substantially from those seeking in-person treatment.

Support for the long-term efficacy of web-based EFT treatment has recently emerged in a 2-year follow-up of a web-based trial for food cravings [59]. The treatment group participants completed a self-paced web-based EFT treatment program comprising 7 modules throughout the 8-week intervention period, and a wait list group also completed the EFT web-based intervention following the end of the intervention period. From preintervention to immediately postintervention and 2-year follow-up, scores significantly improved for food cravings (−28.2%), power of food (−26.7%), restraint (+13.4%), depression (−12.3%), anxiety (−23.3%), and somatic symptoms (−10.6%). Significant improvements were also seen in carbohydrates and fast food cravings between 6 months and 2 years. Findings suggest that ongoing treatment for cravings for desirable food was not required following the 8-week web-based EFT intervention.

The physiological mechanisms of action underlying EFT have been elucidated in several studies. A triple blind RCT compared a single hour-long session of EFT with both talk therapy and rest [50]. Measures included psychological symptoms of anxiety and depression and biological assessment of cortisol, the stress hormone. The study found that psychological distress dropped by more than twice as much in the EFT group as it did in the other two groups. Cortisol levels declined significantly more in the EFT group. Another study examined both cortisol and immunoglobulin levels in participants receiving EFT over the course of a weeklong workshop [60]. A reduction was found in baseline cortisol of 37% and increased synthesis of immunoglobulins by 113% as well as improvements in a range of other physiological markers of general health. A study of pregnant women also found significant decreases in cortisol and increases in immunoglobulins after EFT treatment [61]. In addition, an RCT of veterans with clinical levels of PTSD found a significant reduction of 53% in symptoms such as flashbacks, nightmares, and hypervigilance. Participants received 10 EFT sessions, and their gene expression was measured before and after treatment. Significant upregulation was found in 6 genes related primarily to immunity and suppression of inflammation [62]. A similar study found regulated expression of 2 microRNAs associated with depression [63].

A criticism of EFT is that because it borrows elements from established therapies such as exposure and cognitive therapy, its acupressure component may be no more than placebo. A total of 6 dismantling or component studies rigorously tested this hypothesis and all found that acupoint tapping did indeed enhance treatment results. A review of these studies reported the same effect [64].

### This Study

**Figure 2:**
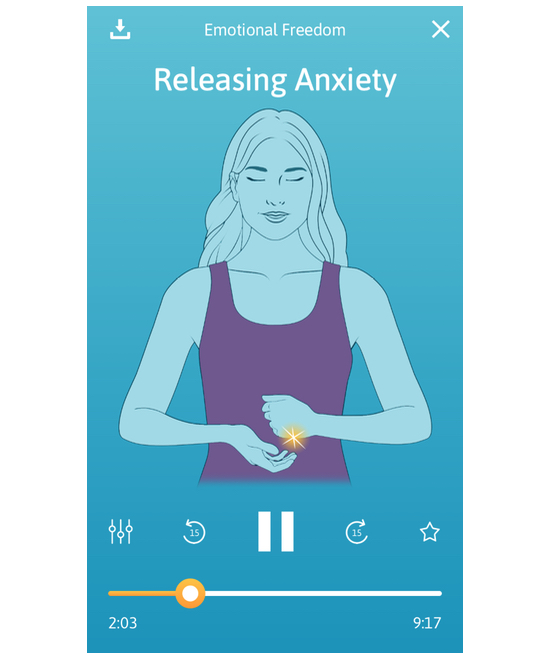
Acupoints are highlighted on 9 acupoints on the face and body. The side of the hand is highlighted in this image.

The Tapping Solution App recorded one million user sessions 12 months following its launch. The Tapping Solution App offers a suite of more than 220 guided tapping meditations, with category titles such as “Emotional Freedom,” “Fears and Phobias,” and “Sleep Support.” Each audio track (≤10 min in duration) guides users through a tapping sequence targeting a particular problem. The content was designed and recorded by a practitioner certified in Clinical EFT by EFT Universe, one of the largest EFT training organizations in the world. The scientific advisory committee for the app included researchers who had collectively published over 40 clinical trials, meta-analyses, and systematic reviews of EFT. The app is free to download from iOS (Apple) and Android (Google Play) platforms, with a paid version available that contains additional content and a lifetime subscription to updates. App features include a personalized dashboard with motivational quotes and a progress tracker, SUD self-assessments at the beginning and end of each tapping meditation, and a download option for offline activity. A visual acupoint graphic (Figure 2) is also presented in each session that highlights the acupoints on the face and body (as previously displayed in Figure 1).

This study sought to evaluate the impact of The Tapping Solution App on intensity self-ratings of anxiety and stress in a large sample of app users. Since studies have found large initial gains from Clinical EFT interventions as symptoms drop rapidly [31], it was hypothesized that a significant reduction in app users’ emotional intensity ratings would be found across app meditations.

## Methods

### Participants and Procedure

This study is a cross-sectional analysis of app user self-ratings. The sample comprised 270,461 app users aged between 18 and 65 years and above who had self-selected and downloaded The Tapping Solution App for use on a mobile device across 12 months from October 2018. The sample consisted of 81.9% (221,508/270,461) women and 18.1% (48,953/270,461) men. Demographics by age and sex are shown in Table 1. Approximately half the app users (135,771/270,461, 50.20%) were located in the United States, followed by the United Kingdom (36,512/270,461, 13.50%), Canada (34,078/270,461, 12.60%), and Australia (27,857/270,461, 10.30%). In terms of device use, 85.50% (324,929/380,034) of app sessions were completed on a smartphone and 14.50% (55,105/380,034) on a tablet device. Participants provided informed consent when agreeing to a statement in the terms and conditions of The Tapping Solution App, which stated that their anonymized data would be used for research purposes. This study was not approved by an institutional review board or ethics committee because the data set that was analyzed consisted of existing third-party data that were deidentified [65].

**Table 1:** Demographics of app users by age and gender (N=270,461).

Demographics		Women, n (%)	Men, n (%)
**Age (years)**			
	18-24	9737 (3.60)	3786 (1.40)
	25-34	28,669 (10.60)	7843 (2.90)
	35-44	45,708 (16.90)	9737 (3.60)
	45-54	55,174 (20.40)	10,548 (3.90)
	55-64	49,765 (18.40)	8655 (3.20)
	≥65	32,726 (12.10)	8384 (3.10)
Total		221,508 (81.90)	48,953 (18.10)

Before each session, app users were advised to consult a doctor regarding any issue relating to a psychological or physiological symptom that required medical attention. Furthermore, the terms and conditions of app use stated that the content provided in the app did not substitute for advice, diagnosis, or treatment from a qualified health care professional. Participants received weekly email updates from one of the app developers who encouraged the use of various app session categories.

### Anxiety App Sessions

The word search function was used within The Tapping Solution App to identify app meditations related to anxiety. In line with criteria from the anxiety app meta-review by Sucala et al [21], app meditation titles containing the words “anxiety,” “worry,” “panic attack,” “social anxiety,” and “fear” (ie, symptoms of generalized anxiety disorder) were used in the analyses. A total of 12 app meditations were identified, which included “Releasing Anxiety,” “Turn Your Day Around—Tapping for Anxiety, Tap and Breathe,” and “Releasing Anxiety in the Breath.”

### Stress App Sessions

The word search function within the app was used to identify app meditations designed to target psychological stress symptoms. A total of 11 app meditation titles that referenced *stress* were identified, including “I’m Stressed About the World,” “Nervous Tension & Stress Release,” and “Releasing Evening Stress.”

### Emotional Intensity Indicator

The SUD scale [32] provided a measure of emotional intensity. Psychological symptoms of anxiety and stress were self-rated by app users on a scale of 0 to 10 (0=*no distress at all* to 10=*worst distress imaginable*) before and after app sessions. This rating was represented on the app using a built-in Visual Analog Scale [66], in which users slid a dot along a visual scale to indicate their symptom intensity rating. Users provided 2 SUD scores: one at the start of the session and another on completion of the tapping meditation. The average time between pre– and post–app session ratings was approximately 10 min. Wolpe [67] developed the SUD scale for use with World War II veterans to measure the emotional impact of traumatic events. Increased SUD scores are associated with heightened arousal of the sympathetic nervous system [68]. SUD ratings are correlated with heart rate, respiratory rate, and galvanic skin response [69]. When interventions lower SUD levels, physiological signs of stress are reversed [70].

### The App Intervention

An example of the app interface during a session is shown in Figure 2. Upon opening the app, users were provided with a short topic summary overview. For example, the *Releasing Anxiety* session description states the session purpose:

Anxiety is not just felt in our minds but with our whole body, which is why using a technique like tapping that uses the mind and body is so powerful. Anxiety often appears when we are worried about the future and feel disconnected from the present moment. Use this tapping meditation to begin to rewire your brain to release anxiety and stress and allow things to be easy.

Session progress (time display in minutes and seconds) was visible for app users throughout each session (Figure 2).

## Results

**Figure 3:**
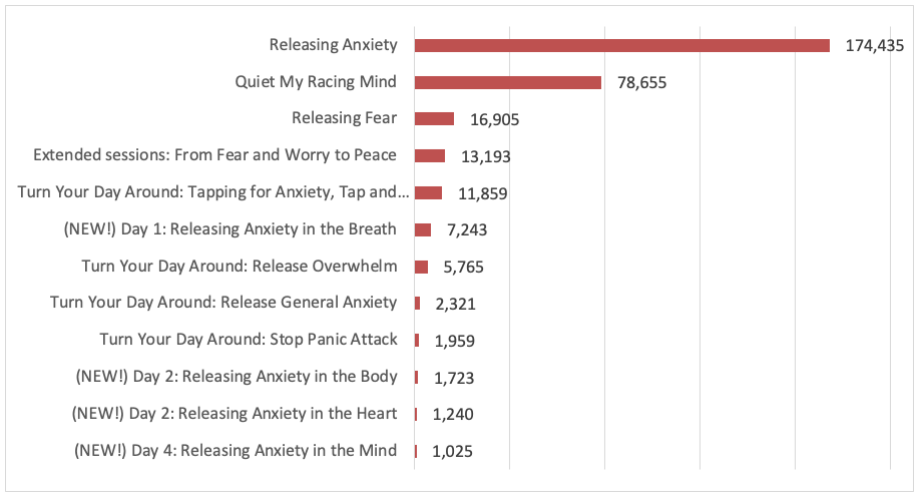
Anxiety app meditation by title and total completed plays (n=316,323).

**Figure 4:**
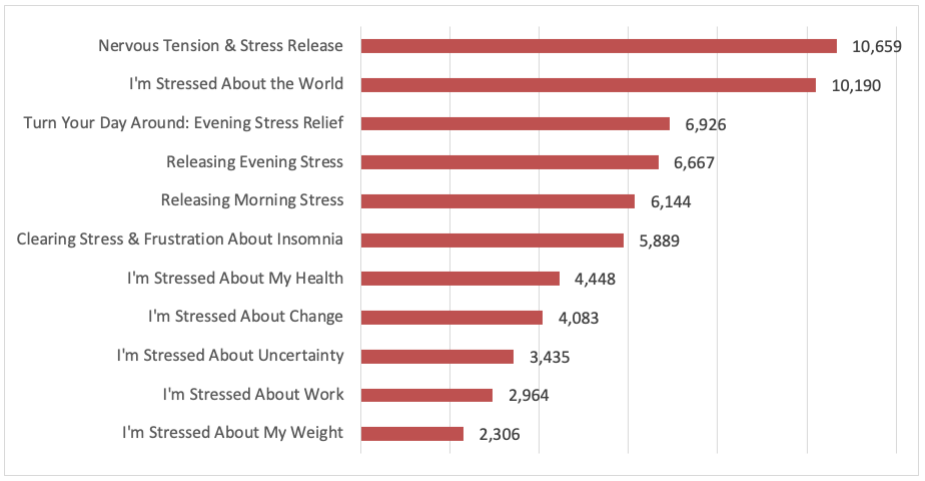
Stress app meditation by title and total completed plays (n=63,711).

A total of 23 meditations available on The Tapping Solution App between October 2018 and October 2019 were identified. App session intensity reports for the 23 meditations, comprising 380,034 completed session plays, were uploaded from Google Analytics and imported to SPSS version 26 for analyses. The completed plays for 12 anxiety meditations ranged from 1025 for “Releasing Anxiety in the Mind” to 174,433 for “Releasing Anxiety.” Completed plays for 11 stress app meditations ranged from 2306 for “I’m Stressed About My Weight” to 10,659 for “Nervous Tension & Stress Release.” Figures 3 and 4 display the 12 anxiety meditations and 11 stress meditations by volume of completed plays and change in the net intensity rating.

Weighted means and SDs for user self-ratings across the anxiety and stress app meditations based on completed plays are displayed in Table 2. Across the 12 anxiety meditations, the difference in emotional intensity ratings from presession (mean 6.66, SD 0.25) to postsession (mean 3.75, SD 0.30) was statistically significant, t_316,322_=−6009.64, *P*<.001, two-tailed, and this was a very large effect size (d=3.71). On average, emotional intensity ratings improved by −2.91 (95% CI −2.92 to −2.91) following app use. Across the 11 stress meditations, the difference in emotional intensity ratings for presession (mean 6.91, SD 0.48) and postsession (mean 3.83, SD 0.54) was statistically significant, t_63,710_=−4455.81, *P*<.001, two-tailed, and a very large effect size (d=6.02). On average, emotional intensity ratings improved by −3.08 (95% CI −3.08 to −3.07) following app use.

**Table 2:** User self-ratings results for anxiety and stress app meditations based on completed app session plays (N=380,034).

App meditation		Total plays, n	Mean (SD)	Minimum score	Maximum score	Change (%)	*P* value
**Anxiety (n=12)**		316,323					
	Presession		6.66 (0.25)	5.73	7.48	N/A^a^	N/A
	Postsession		3.75 (0.30)	3.10	4.54	N/A	N/A
	Net intensity change		2.91 (0.27)	2.47	3.35	−29.13	<.001
**Stress (n=11)**		63,711					
	Presession		6.91 (0.48)	6.19	7.80	N/A	N/A
	Postsession		3.83 (0.54)	2.94	4.60	N/A	N/A
	Net intensity change		3.08 (0.17)	2.87	3.37	−30.80	<.001

^a^N/A: not applicable.

## Discussion

### Principal Findings

Given the staggering volume of mHealth apps available for download on smartphones or mobile devices, research examining the effectiveness of intervention-based mHealth apps is critical. This study aimed to provide a preliminary review of the impact of The Tapping Solution App on psychological distress ratings in a sample of 270,461 app users. Changes in emotional intensity ratings were assessed across 23 anxiety and stress-based app meditations using data from 380,034 completed app plays collated over 12 months. As hypothesized, a significant reduction in app users’ emotional intensity ratings was found across app meditations. Presession to postsession results indicated that emotional intensity ratings dropped an average of 29.13% (*P*<.001) for the anxiety meditations and 30.80% (*P*<.001) for the stress meditations. The current results offer preliminary evidence to support the immediate and large effect of The Tapping Solution App in improving ratings of psychological distress in app users.

The results of this study are consistent with a large body of work that has found EFT to be efficacious in the reduction of symptoms of anxiety and psychological distress [35,47,71]. In the RCT of Church et al [50], statistically significant improvements in subjective reports of anxiety (−58.34%) were found following a brief 50-min EFT session. In this study, mean emotional intensity ratings improved between 29.1% and 30.8% following brief app-delivered tapping sessions. Although levels of psychological distress were measured using self-reported user ratings in this study, the results suggest evidence of statistically significant differences between presession and postsession for self-ratings of psychological distress following app use. The results are in line with electroencephalogram studies of EFT that have measured extensive changes in the activation of neural networks after treatment. These include the suppression of the brain-wave frequencies of anxiety and distress and expansion of those associated with healing and *flow* states [72-74]. Our findings also support previous research that has reported large initial gains from Clinical EFT intervention as symptoms of psychological distress drop rapidly and within highly compressed time frames [31].

Studies that assess brief single-session EFT interventions are more relevant to the study of an app than EFT delivered as traditional psychotherapy. Traditional ongoing psychotherapy has positive effects that may be attributed in part to therapy duration and other factors (eg, the supportive environment, face-to-face demand characteristics) [75]. However, brief single-session administration of EFT closely matches the short app session duration and the single-session use evident in the current user sample. In this study, 270,461 app users completed 380,034 app sessions across a 12-month period. This equates to an average of 1.4 completed plays per app user, which poses a significant question regarding app session repeat usage. It may be that the regular email update provided by the app developer led users’ attention to alternative app sessions within their growing app session repertoire. However, this user aspect was outside the scope of the present preliminary review. Notwithstanding, the results of this study provide strong evidence that emotional intensity ratings immediately improved following a single time, or at least brief, EFT meditation app session of 10 min (or less) in duration.

Other potential issues should be considered in the evaluation of mHealth apps. Individuals who choose app intervention in place of professional health care may find their symptoms of anxiety and/or psychological stress intensify [76]. As a result, some app users may not seek additional therapeutic support, especially if they consider the app treatment to be ineffective [77]. mHealth apps as a therapeutic resource can also present challenges for treatment adherence. In the case of meditation-style techniques, the self-administration aspect of therapy may present difficulties for individuals with minimal meditation experience [12]. However, in the case of The Tapping Solution App, the verbal and visual guidance provided during each session adheres to the principles of Clinical EFT, which can assist even novice meditators. It is therefore recommended that mHealth app development be viewed as an adjunct to professional psychological services. Furthermore, although there is little evidence to suggest negative effects of meditation-based techniques [78], some studies have identified antisocial behavior, reduced emotional stability following meditation, and depersonalization following meditation therapy [79-81]. Emotional responses of fear, dread, and terror have also been reported following personal meditation practice [75]. Although adverse emotional responses to meditation-based apps are unlikely, this research reinforces the importance of high-level examinations of mHealth app efficacy.

### Methodological Issues

As with many web-based surveys, the current large convenience sample comprising app users was determined by self-selection rather than probability sampling, which can lead to biased estimates [82]. Current findings, therefore, remain specific to the self-selected users of The Tapping Solution App. Accordingly, it is important to note that participants may have presented higher levels of motivation than the general population and had previous meditation experience, which could have influenced the observed improvements. In addition, limited app user demographic variables were measured in this study. Future assessment of a range of demographic characteristics, such as socioeconomic factors and previous meditation experience, will help to delineate mHealth app user samples. In line with the recommendations by Firth et al [24], there is a need for research to examine specific populations (eg, anxiety disorders) to help delineate which user groups benefit the most from app-delivered interventions.

Furthermore, the assessment of psychological distress was based on self-reported emotional intensity ratings rather than empirical or clinician-rated psychological measures. Without diagnostic pretreatment and posttreatment assessments, it was not possible to determine the proportion of current users with clinical levels of anxiety disorders. This in turn has limited the generalizability of the findings to a nonclinical population. Finally, the current research did not control for user expectancy effects, the nonspecific effect of any treatment [50], or other potential treatment effects such as environment and frequency of app use. It is important to note that although some investigators were certified and proponents of the EFT method, the statistician and other investigators were not.

### Future Directions

Since global levels of psychological stress are on the rise [1,3], an efficacious and convenient source of unlimited anxiety and stress management resources is needed. The burgeoning field of mHealth offers a dynamic platform for mental health management opportunities. mHealth apps can help facilitate the use of consumer personal data for academic research purposes. This is particularly important because novel data donation is largely supported by individuals when data collection is for research purposes that can benefit individual health [83].

Although the current results suggest that The Tapping Solution App reduced app users’ self-rated emotional intensity relating to anxiety and stress, these findings are preliminary. Further examination of the app as an intervention tool using controls is required, including feasibility, efficacy, and longitudinal research data on app efficacy. In particular, future empirical assessment could align with the proposed frameworks to help investigate technology in health care. For example, Mohr et al [20] proposed the Continuous Evaluation of Evolving Behavioral Intervention Technologies framework as a timely and efficient alternative to RCTs. Their statistical evaluation of app efficacy can be implemented throughout clinical testing and can accommodate changing app versions [21].

As with any mental health intervention, mHealth apps may be more effective for some individuals. It is possible that the mental health characteristics of those seeking web-based treatment may differ substantially from those seeking in-person treatment [58]. Therefore, studies that focus on defining patient groups that benefit most from smartphone apps (ie, treatment for issues such as elevated anxiety) will contribute much needed insight into the field of mHealth app development. Future research could investigate the reasons for user attrition within app interventions. Such insight would help inform more engaging and effective app design in the future [24]. From a clinical perspective, the American Psychiatric Association has an App Evaluation Model available to members [84]. This resource provides a hierarchical rating system and rubric designed to assist the evaluation of mental health apps and guide app recommendations for clinical patients.

The assessment of physiological arousal is another promising area for mHealth technology. Smartphone technology offers the potential to combine stress reduction app interventions with biofeedback in mHealth psychological care [13,85]. Affective states can be assessed together with physiological measures, such as heart rate variability (HRV) and cortisol levels. Such technological assessment would help to define the benefits of EFT-based app intervention and extend previous research that has identified the effect of EFT on measures of heart rate and cortisol [50,60].

Future research could also explore the functional aspects of mHealth app design that increase user engagement and therefore app efficacy in treating psychological distress [24]. Several features of the mHealth platform are thought to increase engagement with therapeutic protocols, including the provision of visual aids and interactive rating functions [85,86]. The Tapping Solution App provided both functions, together with strong auditory features. In each app session, a female or male voice provided guided instruction to the sounds of waterfalls and rhythmic background music. Studies in meditation research have hypothesized that the therapeutic effect is a result of feeling relaxed, which decreases physiological arousal [87,88]. Therefore, efficacy studies examining app sensory features may help guide future app development with benefits for app user engagement.

Apps provide researchers with an opportunity to gather data quickly from large populations. This has the potential to shorten the *translational gap* between discovery and the availability of effective therapies to patients, estimated by several studies to an average of 17 years [89]. Apps also increase the pool of available subjects exponentially; most efficacy studies of psychotherapy rely on trials with fewer than 30 participants per group [46].

Finally, the role of mHealth apps to support clinician-administered evaluations and validated assessments (eg, Beck Anxiety Inventory [90]) is another important future area of study. Although apps could be recommended as part of an overall treatment plan, it is important to recognize that it may be unsafe for patients with severe psychopathology to use apps outside of the clinical setting because of the risk of unsupervised abreactions.

## Abbreviations

EFT: Emotional Freedom Techniques
mHealth: mobile health
PTSD: posttraumatic stress disorder
RCT: randomized controlled trial
SUD: Subjective Units of Distress
